# Early Life Exposure to Antibiotics and Autism Spectrum Disorders: A Systematic Review

**DOI:** 10.1007/s10803-019-04093-y

**Published:** 2019-06-08

**Authors:** Jan Łukasik, Bernadeta Patro-Gołąb, Andrea Horvath, Ruth Baron, Hania Szajewska, the SAWANTI Working Group, Ruth Baron, Isolde Besseling van der Vaart, Dorota Gieruszczak-Białek, Andrea Horvath, Jan Łukasik, Maciej Kołodziej, Bernadeta Patro-Gołąb, Małgorzata Pieścik-Lech, Jaap Seidell, Agata Skórka, Hania Szajewska, Meron Taye, Joanne Ujcic, Arnoud Verhoeff

**Affiliations:** 1 Department of Paediatrics, The Medical University of Warsaw, Żwirki i Wigury 63A, 02-091 Warsaw, Poland; 2 Sarphati Amsterdam, Amsterdam, The Netherlands

**Keywords:** Autism, Antibiotics, Early life, Risk factors

## Abstract

We systematically reviewed evidence from observational studies on the associations between autism spectrum disorders (ASD) and early-life antibiotic exposure. Eleven articles were included in the review. Prenatal antibiotic exposure was associated with a slightly increased risk of ASD in two cohort studies on overlapping populations and in one case–control study; in three other case–control studies, no significant association was found. One cohort study found a slightly reduced risk of ASD after postnatal antibiotic exposure, while two other cohort studies on overlapping populations and three case–control studies reported an increased risk. Meta-analysis of the eligible studies showed no significant associations. Current data are conflicting and do not conclusively support the hypothesis that early-life antibiotic exposure is associated with subsequent ASD development.

**Electronic supplementary material:**

The online version of this article (10.1007/s10803-019-04093-y) contains supplementary material, which is available to authorized users.

Autism spectrum disorders (ASD) are a group of neurodevelopmental conditions characterized by persistent deficits in social communication and restricted, repetitive patterns of behavior, interests, or activities, beginning in the developmental period (American Psychiatric Association 2013). Currently used term ‘ASD’ is an umbrella diagnosis for a group of conditions, namely: autistic disorder, Asperger syndrome, childhood disintegrative disorder and pervasive developmental disorder-not otherwise specified (PDD-NOS). The World Health Organization estimates that approximately one in 160 children has ASD (World Health Organization 2017). However, according to recent studies, the current prevalence of ASD in certain communities may fall between 1 and 2% (Christensen et al. 2016; Brugha et al. 2011). Moreover, the prevalence of autism has steadily risen during the last decades (Lai et al. 2014). The etiology of ASD is heterogeneous (Howes et al. 2018). Multiple genetic factors (Miles 2011), a variety of environmental factors (Hertz-Picciotto et al. 2018), and a complex interplay between these factors (Chaste and Leboyer 2012) have been proposed and extensively studied. A recent rise in the prevalence of ASD suggests a major role of the environment. Since the symptoms of ASD typically occur during early childhood, potential causative factors are most likely to act during the prenatal and early postnatal periods.

One of the investigated risk factors for ASD is exposure to antibiotics during pregnancy and childhood. It has been suggested that the intestinal microbiota affects brain function and human behavior, through the so-called microbiome-gut-brain axis (Cryan and Dinan 2012). Antibiotics are one of the factors known to disturb the composition of the microbiota in infancy (Yassour et al. 2016). Additionally, a number of studies have confirmed that individuals with ASD have different intestinal microbiome compositions compared to those of healthy individuals (Kraneveld et al. 2016). In regard to potential effects of antibiotics on ASD, improvement of ASD symptoms after antibiotic use was reported in two case studies (Sandler et al. 2000; Rodakis 2015). However, high use of different antibiotics in children who subsequently developed autism was shown in a number of observational studies (Fallon 2005; Niehus and Lord 2006; Bittker and Bell 2018). Hypothetically, these observed effects could be attributable to microbiota alterations, triggering a disturbed immune response and the release of cytokines, thus, affecting the function of the central nervous system (de Theije et al. 2011). Recently, a number of observational studies have assessed a possible association of early-life antibiotic exposure and subsequent development of ASD. Here we aimed to systematically document available evidence on the association between early life antibiotic exposure and the prevalence of ASD later in in childhood.

## Methods

### Inclusion/Exclusion Criteria for the Review

Cohort studies, case–control studies, and cross-sectional studies were eligible for inclusion in this review. The included studies needed to investigate an association between pre- and/or postnatal antibiotic exposure and subsequent diagnosis of ASD. We included studies in which women during any trimester of pregnancy or infants and/or children underwent antibiotic treatment. We focused on early-life antibiotic exposure that preceded a diagnosis of ASD, which is usually established after the second year of life (Mandell et al. 2005). Studies on antibiotic exposure in breastfeeding mothers and subsequent risk of ASD in their children were not included. Since the hypothesized link between ASD and antibiotic use is based on the aforementioned microbiome-gut-brain axis mechanism, only studies which reported data on systemic and/or oral antibiotic therapy were taken into an account. Any antibiotic types and doses, as well as any treatment durations and indications were admissible, as long as the therapy fulfilled the aforementioned criteria. Studies that reported data on antibiotic use collectively (e.g., along with other medications) or that did not report numerical data were excluded. Studies in which the data on antibiotic use was collected only for the purpose of baseline characteristic of participants, with no analysis of the association between antibiotic use and ASD were also excluded.

Our outcome of interest was the diagnosis of ASD during childhood. Studies in which the diagnosis was made according to established criteria, such as those described in the Diagnostic and Statistical Manual of Mental Disorders-V (American Psychiatric Association 2013), as well as studies in which ASD was reported by parents, caregivers or doctors without any described specific criteria, were available for inclusion. Studies that reported data on ASD only collectively with other neurodevelopmental disorders (e.g., together with attention-deficit hyperactivity disorder), were excluded. Only studies that compared children with ASD up to 18 years of age to generally healthy children without this diagnosis were included.

### Search Methods

A systematic literature search was performed on 28th of August 2018, with no language or publication date restrictions. The databases searched were PubMed, Embase, and PsycINFO. After drafting the first version of the manuscript, we ran a search update on the 11th of December 2018. The full search strategies for PubMed and Embase are presented in Online Resource 1. Additionally, reference lists of identified observational studies and relevant review articles were manually screened.

### Study Selection

Three authors/reviewers (AH, BPG, JŁ) independently screened the title of each study identified with the search strategy as well as the abstracts of potentially relevant articles. Subsequently, the full text for each study potentially relevant after abstract screening, as well as that for studies of unclear relevance, was retrieved. Each author independently assessed the eligibility of the articles and, in cases of a disagreement, resolved differences by discussion.

### Data Extraction and Risk of Bias Assessment

Data were extracted with the use of a standard data extraction form. The extracted data included study year, country, design, population, definition of exposure, definitions of cases and controls (for case–control studies), definition of outcome (for cohort studies), results, confounding factors, and data collection methods. We extracted and reported all the data using the same terminology as the authors of the original articles.

Risk of bias was assessed using the Newcastle–Ottawa Scale (NOS) (Wells et al. 2011), in which the reviewers assign stars in all predefined bias domains. In the comparability domain the reviewers have to assign 0, 1, or 2 stars on the basis of the number and types of confounding factors controlled for. Multiple important confounding factors may play a role in studies on the antibiotic-ASD association, including those related to different demographics, pregnancy-related complications, mothers’ obstetrical histories, child characteristics at birth, infections, environmental exposures, and healthcare use (Lyall et al. 2017). The NOS requires the reviewers to assign stars depending on two chosen, most important factors. After the literature search and discussion, we found no rationale to decide whether any of the above-mentioned factors was more important than the others. Therefore, we decided to assign zero stars to studies in which no confounding factors were controlled for or the adjustment was unclear, and two stars to studies which controlled for any confounding factors. Additionally, for informative purposes, we present confounders identified in each study in a separate table. We decided to present risk of bias only descriptively and no collective score for the included studies was counted.

### Data Analysis

A meta-analysis of the findings was performed if at least two studies of the same type (cohort, case–control) reported the adjusted odds ratios (aOR) or adjusted hazard ratios (aHR) of ASD being diagnosed in participants exposed to antibiotics during the same life period (pre- or postnatally). We performed the meta-analyses using the Review Manager 5.3 software by the Cochrane Collaboration. Generic inverse variance with the random effects model was used. Additionally, the fixed effects model was applied to see how it would influence the results. To enable a graphic presentation of the findings in studies which didn’t report OR or HR, we calculated crude ORs/HRs and 95% confidence intervals (95% CI) of ASD being diagnosed after antibiotic exposure, provided the data were sufficient.

## Results

### Overall Characteristics

**Fig. 1:**
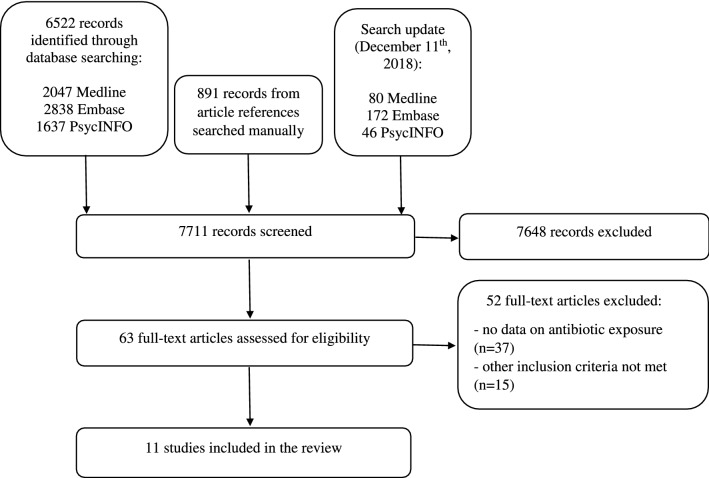
Flow chart diagram

In total, we identified 6820 records by the database search and 891 records by reference screening. After exclusion of duplicates and title and abstract screening, the full texts of 63 articles were assessed for eligibility. Thirty-seven observational studies on risk factors for autism were excluded because they did not report antibiotic exposure. Reasons for exclusion of the remaining 15 studies are listed in Online Resource 2. After full-text assessment, 11 articles ultimately met the inclusion criteria for this review. The flow diagram of the selection process is presented in Fig. 1.

Online Resources 3 and 4 present characteristics and main results of the included studies. Four of those studies were cohort studies, and seven of them were case–control studies. Among the included cohort studies, three Danish studies (Atladottir et al. 2012; Wimberley et al. 2018; Axelsson et al. 2019) were performed in overlapping populations. Five studies examined prenatal antibiotic exposure, five assessed early childhood antibiotic exposure and 1 study examined both types of exposure (Wimberley et al. 2018). In the majority of included studies, use of any antibiotic, for any indication and duration and at any dose within the defined time period, was analyzed. Additionally, all of the cohort studies and two of the case–control studies included a number of secondary/sensitivity analyses, taking antibiotic type, spectrum, timing, number of courses, and/or indication into account.

Overall, three out of four included cohort studies earned a maximum number of stars, and only one study lost one star in one of the NOS domains. In contrast, all of the case–control studies carried a considerable amount of bias, mostly concerning case definitions, control for confounding factors, and ascertainment of exposure. Details of risk of bias assessment for cohort and case–control studies, respectively, are presented in Online Resource 5. Additionally, we listed all the confounding factors used in adjusted analyses in Online Resource 6.

### Prenatal Antibiotic Exposure

#### Cohort Studies

Prenatal antibiotic exposure as a risk factor for autism was examined in two large Danish cohort studies performed in overlapping populations. The studies involved, respectively: 96,736 children, including 976 with ASD (Atladottir et al. 2012) and 780,547 children, including 9352 with autism (Wimberley et al. 2018). Population of the first study was most likely contained within the population of the second, therefore, we didn’t perform a meta-analysis. The first study reported a borderline significant increase in the risk of ASD after use of any antibiotics anytime during pregnancy (adjusted hazard ratio [aHR] 1.2; 95% confidence interval [CI] 1.0–1.4). A stronger association of prenatal antibiotic exposure with the risk of ASD was reported for the use of sulfonamides anytime during pregnancy (aHR 1.5; 95% CI 1.0–2.2) and use of penicillins during the second (aHR 1.4; CI 1.1–1.8) and third (aHR 1.4; CI 1.0–1.8) trimester. Similar results were observed for infantile autism (defined as ICD-10 code F84.0 assigned to participant), with the strongest association reported for the use of macrolides anytime during the pregnancy (aHR 2.2; 95% CI 1.1–4.4). Confounding factors controlled for in adjusted model were as follows: gender, maternal age, parity, maternal smoking during pregnancy, paternal age, parental psychiatric history, and parents’ educational status.

The second study reported a significant, positive association between exposure to any antibiotic anytime during pregnancy and subsequent risk of autism in the offspring (aHR 1.18; 95% CI 1.13–1.23). The analysis was adjusted only for sex and age. Assessment of this association was not a primary focus of the study, and it was only reported as a secondary analysis in the supplementary materials.

#### Case–Control Studies

**Fig. 2:**
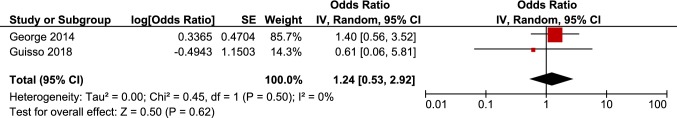
Meta-analysis of case–control studies on prenatal antibiotic exposure

Four of the included case–control studies examined risk of ASD after prenatal antibiotic exposure. Among them, two clinic-based studies (George et al. 2014; Guisso et al. 2018), were available for a meta-analysis. They were conducted in groups of children of 343 (143 with autism) and 314 (136 with ASD), respectively. None of those studies found significant associations between antibiotic use and ASD, with pooled odds ratio from random effect model of 1.24; 95% CI 0.53–2.92 (Fig. 2). In the first study, confounding factors used for the analysis were not described (George et al. 2014), while the second study controlled for a number of perinatal, gestational, and socioeconomic variables (Guisso et al. 2018).

In one study conducted in 288 Polish children including 96 with autism (Mrozek-Budzyn et al. 2013), the results were presented as the percent of cases versus percent of controls exposed to prenatal antibiotics. The univariate model revealed significantly higher exposure to antibiotics in the autism group (17% vs. 2%, p < 0.001). However, for undescribed reasons, the antibiotics were analyzed only collectively with other medications in the multivariate model.

The last study was performed in 415 Swedish children, including 206 with ASD (Isaksson et al. 2017). In the statistical model, autism was regressed on antibiotic use during pregnancy. The data were presented as logistic regression betas in the form of log odds (β = − 0.15, standard error = 0.45, p > 0.05 in regular regression; β = 1.78, standard error = 1.04, p > 0.05 in fixed effects regression) and were reported by the authors as not statistically significant.

**Fig. 3:**
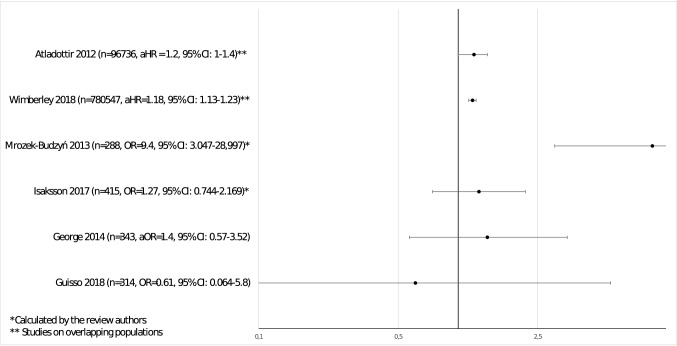
Hazard ratios and odds ratios of ASD in exposed versus unexposed groups—data from studies on prenatal antibiotic use

None of the aforementioned case–control studies investigated type of antibiotic, duration of antibiotic therapy, or its timing during pregnancy. All ORs and HRs for ASD after use of prenatal antibiotics versus non-exposed group are presented graphically in Fig. 3.

### Early Childhood Antibiotic Exposure

#### Cohort Studies

Early childhood antibiotic exposure as a risk factor for autism was examined in three cohort studies. One study performed in 214,834 Canadian children, including 2965 with ASD (Hamad et al. 2018), reported a trend towards a reduced risk of ASD in children exposed to any antibiotics during the first year of life after adjusting for relevant demographic, prenatal, perinatal, and postnatal factors (aHR 0.91; 95% CI 0.84–0.99). Stratifying the analysis by sex and region resulted in a statistically significant association in males (aHR 0.91; 95% CI 0.83–1.00) and in children from urban regions (aHR 0.85; 95% CI 0.77–0.94). In secondary analyses, a borderline significance was found for the association of specific antibiotic groups with ASD, namely penicillin (aHR 0.92; 95% CI 0.84–1.00) and macrolides (aHR 0.87; 95% CI 0.77–0.99). The number of antibiotic courses and duration of therapy were not associated with ASD risk. In additional analysis of sibling-controlled design, antibiotics use was not associated with risk of developing ASD.

Two other cohort studies were nation-wide, register-based studies of the Danish population. Both of these studies derived data from the same registers and covered similar time periods, so their populations largely overlapped. In one study in 677,403 children, including 8267 with autism (Axelsson et al. 2019), a slightly increased risk of autism was observed in children exposed postnatally to penicillin (aHR 1.11, 95% CI 1.04–1.19) as well as to broader-spectrum antibiotics (aHR 1.10, 95% CI 1.04–1.16) compared to children unexposed to antibiotics in the standard model. However, a significant association was no longer observed in the between-within sibling model (aHR 1.05, 95% CI 0.93–1.18 and aHR 1.05, 95% CI 0.95–1.16 for penicillin and broader-spectrum antibiotics, respectively). In another study performed in 780,547 children including 9352 with autism (Wimberley et al. 2018), the risk of autism increased after broad-spectrum antibiotic exposure before the age of 5 (aHR 1.25, 95% CI 1.09–1.42). Moreover, risk of autism was higher after exposure to broad-spectrum and moderate-spectrum antibiotics anytime during childhood (aHR 1.51, 95% CI 1.33–1.72 and aHR 1.22, 95% CI 1.13–1.32, respectively). Both of these studies controlled for a range of potential confounders, including different demographic, pregnancy and neonatal factors, parental psychiatric history, and parity.

**Fig. 4:**
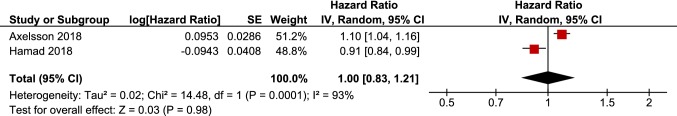
Meta-analysis of cohort studies on postnatal antibiotic exposure—standard model

**Fig. 5:**
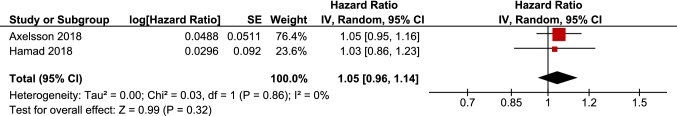
Meta-analysis of cohort studies on postnatal antibiotic exposure—sibling model

Since at least two studies of the same type reported the aHR of ASD after antibiotic exposure, a meta-analysis was feasible. However, the two Danish studies were performed in highly overlapping populations, so pooling of their data was not justifiable. To perform a meta-analysis, we used the data from the 1 of the 2 Danish studies, in which the exposure and its timing were consistent with those in the Canadian study (Hamad et al. 2018). Pooling of the data did not yield any significant association between antibiotic exposure and subsequent diagnosis of ASD, either in the standard model (summary effect estimate 1.00, 95% CI 0.83–1.21) or in the sibling model (summary effect estimate 1.05, 95% CI 0.96–1.14), both in random effects (Figs. 4, 5) and fixed effects analysis.

#### Case–Control Studies

Three case–control studies examined association between ASD and childhood antibiotic exposure. One internet survey-based study performed in 1515 children (1001 with ASD) (Bittker and Bell 2018), which reported the data in the form of logistic regression OR, found a positive association between the number of antibiotic courses taken during the first 2 years of life and subsequent diagnosis of ASD in all performed analyses, including the final multifactor model (aOR 1.083; 95% CI 1.023–1.151). The study controlled for age of the child, ethnicity, maternal education, and region.

Another study performed in a group of 214 children (73 with autism) (Grossi et al. 2018) reported crude ORs of autism in children exposed postnatally to antibiotics compared to unexposed unrelated controls (OR 2.43; 95% CI 0.9–6.52) and unexposed healthy siblings (OR 2.03; 95% CI 0.40–9.1). Additionally, a multivariable modeling of data was performed with use of the Auto-CM artificial neural network—a method that does not generate numerical results but rather provides a map of associations of strength between all variables in the data set. According to the authors’ interpretation, the proximity of ‘early antibiotic use’ to the ‘autism node’ observed on the map may suggest a significant association between the two.

In one case–control study involving 99 children (75 with autism/ASD) (Niehus and Lord 2006), the mean number of antibiotic courses taken before the age of 2 years was significantly higher in the autism/ASD group compared to the control group (6.88 vs. 3.48, p < 0.01). Data provided by the authors were insufficient to calculate ORs.

**Fig. 6:**
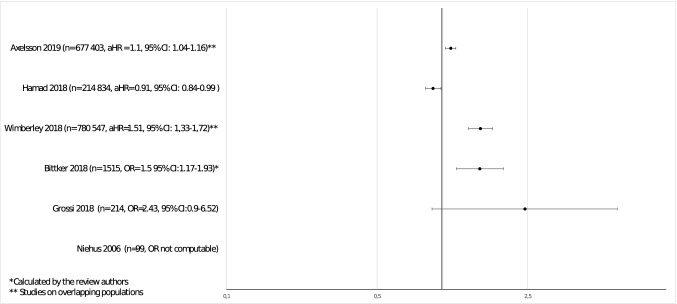
Hazard ratios and odds ratios of ASD in exposed versus unexposed groups—data from studies on postnatal antibiotic use

None of these case–control studies assessed specific types of antibiotics nor the duration of therapy within the analyses. ORs and HRs of ASD in exposed versus unexposed groups for these studies are presented on Fig. 6.

## Discussion

In this review on pre- and postnatal antibiotic exposure and subsequent risk of autism/ASD, a total of 11 observational studies were included. In two identified cohort studies on prenatal antibiotic exposure performed in overlapping populations, a slightly increased risk of ASD/infantile autism after the use of various antibiotics was observed. However, 3 of 4 identified case–control studies did not report any significant association between prenatal antibiotic exposure and autism risk, whereas one reported a positive association based only on univariate analysis. Three identified cohort studies on early childhood antibiotic exposure reported conflicting evidence. In one cohort study, a small trend towards a reduced risk of ASD in infants exposed to various antibiotics in their first year of life was observed, which was not significant in secondary analyses based on sibling-controlled design. In another cohort study, a borderline increased risk of ASD was observed with exposure to certain types of antibiotics in a standard model but no association was observed in a between-within sibling model. Finally, in the third study on a cohort overlapping with the previous one, a significantly higher risk of autism after exposure to various classes of antibiotics was observed. Data from case–control studies on antibiotic exposure during childhood were more consistent. All three of them reported significant, positive associations between antibiotic use and later ASD diagnosis.

To our knowledge, no previous systematic review has examined antibiotic use during pregnancy and early childhood as a potential risk factor for ASD. A number of meta-analyses have reported data on various infections preceding ASD diagnosis (Modabbernia et al. 2017), and some have found significant positive associations with infections occurring during pregnancy (Jiang et al. 2016; Gardener et al. 2009). Additionally, one of those systematic reviews reported increased risk of ASD after prenatal exposure to different medications, including antibiotics, which were, however, not assessed separately (Gardener et al. 2009). In another meta-analysis, no associations between early postnatal infections and subsequent risk of ASD were found (Gardener et al. 2011). On the other hand, some observational studies have found evidence of a higher risk of mental disorders after various infections in older children and in adults (Benros et al. 2011; Kohler et al. 2017). Recently, more scientific attention is being drawn towards effects of early-life antibiotic exposure on long-term health outcomes, including reports of its negative influence on neurocognitive development (Slykerman et al. 2019) and risk of obesity (Miller et al. 2018).

The main strength of this review is its novelty, as no previous systematic review has focused on the association between early-life antibiotic exposure and ASD. Our study utilized a broad search strategy, complemented by an extensive reference hand search, so the possibility of missing important published data is low. A relatively narrow clinical question allowed us to focus in detail on the included studies and enabled a clear and meticulous presentation of findings. However, this review also has considerable limitations. Compared to the number of other potential environmental risk factors for ASD, antibiotic exposure turned out to be rarely assessed (Wang et al. 2017; Modabbernia et al. 2017). Thus, only 11 studies were finally included in the review, the majority of which were of case–control design. After division into groups depending on study type and timing of exposure, small groups of 2 to 4 studies were created. Methods of data reporting in these studies were heterogeneous, further limiting the capacity to perform either qualitative or quantitative synthesis of the data. Moreover, each of the included case–control studies was characterized by a significant potential risk of bias. Another limitation of this study is the inclusion of only published data, creating a risk of publication bias. Finally, we excluded a number of studies in which infections were explored as potential risk factors for ASD. Some types of these infections are almost always treated with antibiotics (e.g., urinary tract infections during pregnancy, severe infections in newborns), so we could have assumed them to be a proxy for antibiotic exposure. However, we decided to use only direct data on exposure.

Results of the included studies do not provide conclusive evidence to support the hypothesis that prenatal antibiotic exposure is associated with ASD. Although a number of positive associations was reported in two large cohorts (Atladottir et al. 2012; Wimberley et al. 2018), their populations were largely overlapping, so they cannot be treated as independent studies for the purpose of this review. Moreover, authors of the study with borderline significant results (Atladottir et al. 2012) emphasize, that within their analysis they made 106 comparisons with no adjustments for multiple testing, so this borderline significant association between prenatal antibiotic exposure and ASD could have been a chance finding. While this study was specifically designed to examine prenatal risk factors of autism and tested for a wide range of potential confounders, the other one focused on postnatal factors and hardly performed any adjustment in the prenatal exposure analysis. Because of that, in our opinion the results of the former study are more meaningful.

On the other hand, all of the case–control studies reported no association between prenatal antibiotic exposure and ASD, except for one that reported higher risk of autism in exposed patients (Mrozek-Budzyn et al. 2013). However, this study was performed in a relatively small group of children, and strangely, its finding concerning antibiotic exposure was not explored in a multivariate analysis. Moreover, compared to the literature (Santos et al. 2010; de Jonge et al. 2014) an unexpectedly low percentage of women in the control group (2.1%) was reported to have used antibiotics during pregnancy, which may suggest a bias within the exposure data.

Results of studies on postnatal antibiotic exposure are conflicting. One cohort study reported a reduced risk of ASD in children after antibiotic use (Hamad et al. 2018). However, since the main model was based only on data from administrative databases, the authors performed secondary analyses based on a sibling-controlled design. In those analyses, antibiotic use was not associated with the risk of developing ASD, hence, the authors concluded that the marginal association observed in the main model was unlikely to be meaningful. In another cohort study (Axelsson et al. 2019), the between-within sibling design analysis did not confirm the findings from the standard design analysis of higher ASD risk after use of certain types of antibiotics. We used data from these two studies to perform a meta-analysis, and its results revealed no significant association. The final included cohort study (Wimberley et al. 2018) reported a positive association between postnatal antibiotic exposure and autism risk. However, since its population largely overlapped with that of the previous cohort, the two cannot be treated as independent studies for the purpose of this systematic review. Besides the lack of a sibling analysis in the latter cohort, the difference in results might have originated from the differences in the exposure assessment. The authors of the latter study defined ‘antibiotic use preceding the diagnosis of autism’ as the antibiotic received before the first diagnosis of autism registered in the national healthcare databases. Median age for the first autism diagnosis in this study was 7.1 years, which is much later than both median age of ASD diagnosis reported in the literature and median age of first developmental concerns in ASD children (Baio et al. 2018). Therefore, even for the subgroup of children exposed to antibiotics before fifth birthday it is unclear, if the symptoms of autism were absent before the first antibiotic administration. In our opinion, this might have introduced considerable bias.

All of the remaining case–control studies reported a positive association between postnatal antibiotic exposure and ASD. All of these studies were characterized by considerable potential sources of bias, including small sample sizes (Grossi et al. 2018; Niehus and Lord 2006), self-selection bias (Niehus and Lord 2006; Bittker and Bell 2018) reflecting differences between those who choose to participate in a study and those who do not, potentially biased control group selection (Grossi et al. 2018; Niehus and Lord 2006), and/or lack of verification if the ASD diagnosis in the case group was made by a professional (Bittker and Bell 2018). Also, in all of these studies, the exposure data were based on parental reports, so a risk of recall bias (Coughlin 1990), especially over-reporting the exposure in ASD groups and under-reporting in control groups, cannot be ruled out.

Data on specific antibiotic types and risk of ASD are also inconsistent. In one study, an increased risk of ASD was reported after prenatal exposure to sulfonamides, penicillins and macrolides, but not cephalosporins (Atladottir et al. 2012). This is consistent with the evidence that macrolides induce distinctive changes in the microbiome composition of children (Korpela et al. 2016). On the other hand, expected microbiota alterations after the use of cephalosporins should be at least as strong as those after use of penicillins, due to cephalosporins’ broader spectrum (Biedermann and Rogler 2015). Accordingly, another study reported a stronger association between ASD and both pre- and postnatal exposure to broad spectrum antibiotics (cephalosporins, tetracycline, trimethoprim-sulfonamide combination, aminoglycosides, and quinolones) compared to moderate-spectrum antibiotics (Wimberley et al. 2018). However, no significant associations were found between autism risk and exposure to narrow-spectrum antibiotics, including macrolides. In two other cohort studies, no significant associations between ASD and individual types of antibiotics were described (Axelsson et al. 2019; Hamad et al. 2018). Individual antibiotic types were analyzed and reported differently in each of the studies included in our systematic review; therefore, a meta-analysis was not possible. According to the available evidence, some patterns of microbiota composition in individuals with ASD have been identified, mostly regarding the abundance of different Clostridia and Bacteroidetes species and an altered Bacteroidetes/Firmicutes ratio (Rosenfeld 2015). Such changes may be induced both by macrolides and broad-spectrum antibiotics (Korpela et al. 2016). On the other hand, microbiota alterations in different individuals might not follow a specific pattern even after use of the same antibiotic (Yassour et al. 2016). Apart from the disturbance of the microbiome-gut-brain axis, to our knowledge no well-documented alternative explanations for the antibiotic-ASD association have been proposed.

A question concerning all of the included studies is the difficulty in differentiating whether the observed effects were due to the antibiotics themselves or were due to the antibiotics acting as a proxy or mediator of an underlying infection. Only two of the included studies controlled for infections occurring during the same period as antibiotic exposure (Hamad et al. 2018; Guisso et al. 2018). None of the included studies investigated differences in risk of autism resulting from specific indications for antibiotic therapy. Three included studies reported a higher risk of autism after maternal respiratory infections (Atladottir et al. 2012; George et al. 2014), fever episodes and influenza (Atladottir et al. 2012), or “infections” in general (Guisso et al. 2018); two studies found significant positive associations between early childhood otitis media episodes and autism (Wimberley et al. 2018; Niehus and Lord 2006). Such associations of prenatal and early-life infections with autism have been documented before (Jiang et al. 2016; Atladottir et al. 2010), and they may also be supported by underlying biological mechanisms (Singh 2009; Ponzio et al. 2007; Rosenhall et al. 1999).

All of the included studies in which the assessment of exposure was based on parental reports collected after diagnosis of ASD have the possibility of recall bias. Knowledge of a variety of potential exposures that are considered as risk factors for ASD is common among parents of children with ASD (Chaidez et al. 2018); thus, they may tend to analyze the previous medical history of their children more and report medication use more often than parents of the control children. This is a similar effect to the one observed after past claims of the supposed vaccination-autism association (Andrews et al. 2002). This may suggest a greater reliability of antibiotic exposure data collected prospectively or derived from medical records.

Yet another problem in the interpretation of possible antibiotic-ASD associations is the fact that, despite stable antibiotic use in Europe and USA in recent years (European Centre for Disease Prevention and Control 2018; Klein et al. 2018), the prevalence of ASD is actually rising in these regions (Lyall et al. 2017). Nevertheless, given the heterogeneous etiology of autism, it is difficult to exclude the possibility that reduced antibiotic use is outweighed by other environmental factors contributing to ASD risk.

## Conclusions

Currently available data from observational studies do not conclusively support the hypothesis that prenatal or postnatal exposure to antibiotics as a group is a risk factor for ASD. However, a portion of the results from large cohort studies may suggest a possible trend towards higher risk of ASD after use of certain types of antibiotics. More studies, most preferably of a prospective cohort design, controlling adequately for potential confounding factors including infections, are needed to verify this association.

## Electronic supplementary material

Below is the link to the electronic supplementary material.

Supplementary material 1 (DOCX 15 kb)

Supplementary material 2 (DOCX 36 kb)

Supplementary material 3 (DOCX 34 kb)

Supplementary material 4 (DOCX 35 kb)

Supplementary material 5 (DOCX 25 kb)

Supplementary material 6 (DOCX 17 kb)
