# Supplementary material for: Early Life Exposure to Antibiotics and Autism Spectrum Disorders: A Systematic Review
Source: J Autism Dev Disord. 2019 Jun 8;49(9):3866–76. doi: 10.1007/s10803-019-04093-y (PMC6667689; doi:10.1007/s10803-019-04093-y)
Supplement: Supplementary file 1 — Supplementary material 1 (DOCX 15 kb) [file 10803_2019_4093_MOESM1_ESM.docx]

Online Resource 1. Search strategy for Medline (Pubmed).

| #1 | “Risk Factors”[Mesh] OR risk factors [All fields] OR "Anti-Bacterial Agents"[Mesh] OR "Anti-Bacterial Agents"[Pharmacological Action] OR "Penicillins"[Mesh] OR antibacterial*[tiab] OR anti-bacterial*[tiab] OR antibiotic*[tiab] OR antimicrobial[tiab] OR anti-microbial[tiab] OR anti-infective[tiab] OR antiinfective[tiab] OR penicillin*[tiab] OR macrolide*[tiab] OR phenethicillin*[tiab] OR clavulanat*[tiab] OR flucloxacillin*[tiab] OR amoxicillin*[tiab] |
| --- | --- |
| #2 | "Child Development Disorders, Pervasive"[Mesh] OR pervasive developmental disorder*[tiab] OR autis*[tiab] OR pdd[tiab] OR childhood schizophrenia[tiab] OR kanner[tiab] OR asperger[tiab] |
| #3 | “Child”[Mesh] OR child*[tiab] OR “Infant”[Mesh] OR infan*[tiab] OR newborn*[tiab] OR baby*[tiab] OR babies*[tiab] OR toddler*[tiab] OR Pregnancy[Mesh] OR pregnan*[tiab] OR gestat*[tiab] OR prenatal*[tiab] OR perinatal*[tiab] OR postnatal*[tiab] OR antenatal*[tiab] |
| #4 | #1 AND #2 AND #3 |

Online Resource 2. Search strategy for Embase

| #1 | 'risk factor'/exp OR 'risk factor*':ab,ti OR 'antibiotic agent'/exp OR 'antibiotic*':ab,ti OR 'antiinfect*':ab,ti OR 'antimicrob*':ab,ti OR 'anti-infect*':ab,ti OR 'anti-microb*':ab,ti OR 'antibact*':ab,ti OR 'anti-bact*':ab,ti |
| --- | --- |
| #2 | 'autism'/exp OR 'autis*':ab,ti OR 'pervasive developmental disorder*':ab,ti OR 'pdd':ab,ti OR 'kanner':ab,ti OR 'asperger':ab,ti OR 'childhood schizophrenia':ab,ti |
| #3 | ‘child'/exp OR 'pregnancy'/exp OR 'child*':ab,ti OR 'toddler*':ab,ti OR 'infan*':ab,ti OR 'newborn*':ab,ti OR 'baby':ab,ti OR 'babies':ab,ti OR 'pregnan*':ab,ti OR 'gestat*':ab,ti OR 'prenatal*':ab,ti OR 'perinatal*':ab,ti OR 'postnatal*' OR 'antenatal*' |
| #4 | #1 AND #2 AND #3 |
