# Supplementary material for: Early Life Exposure to Antibiotics and Autism Spectrum Disorders: A Systematic Review
Source: J Autism Dev Disord. 2019 Jun 8;49(9):3866–76. doi: 10.1007/s10803-019-04093-y (PMC6667689; doi:10.1007/s10803-019-04093-y)
Supplement: Supplementary file 2 — Supplementary material 2 (DOCX 36 kb) [file 10803_2019_4093_MOESM2_ESM.docx]

| **Study ID** | **Study Population** | **Type of Exposure** | **Definition of controls** | **Definition of cases** | **Results** | **Additional comments** |
| --- | --- | --- | --- | --- | --- | --- |
| Bittker 2018  USA | - Children with ASD n=1001  - Controls n=514  - Participants age: 3-12 years | - Postnatal antibiotic exposure during first 2 years of life  - Data on exposure obtained from parents (web survey)  - Indication for antibiotic therapy not reported | Children without ASD, ADHD, attention deﬁcit disorder, sensory processing disorder, apraxia, or other neurologic condition, according to survey respondent. | Children with ASD, autism, Asperger’s syndrome, or PDD-NOS, diagnosed by a professional, according to survey respondent. Children with a genetic condition  of high ASD penetrance were excluded. | The odds of having ASD, presented as logistic regression ORs and aOR for antibiotic exposure scaled depending on mean number of antibiotic courses taken during the first 2 years of life:  - logistic regression analysis: OR 1.138 (95% CI 1.083-1.201), aOR 1.103 (95% CI 1.046-1.168)  - multifactor model: aOR 1.083 (95% CI 1.023-1.151) |  |
| George 2014  India | - Children with autism n = 143  - Controls n = 200  - Participants age: 2-6 years | - Prenatal antibiotic exposure.  - Data on exposure obtained from mothers/caregivers (structured interview)  - Indication for antibiotic therapy not reported | Children without autism, in the same age group, recruited from the well-baby/immunization clinic. | Children (attending autism clinic of Child Development Centre) who had CARS score of ≥30. | The odds of having autism in children exposed to prenatal antibiotic therapy as compared to unexposed:  - crude OR 2.47 (95% CI: 1.195-5.129)  - aOR 1.40 (95% CI: 0.57–3.52) |  |
| Guisso 2018  Lebanon | - Children with ASD n = 136  - Controls n = 178  - Participants age: 2 - 18 years | - Prenatal antibiotic exposure.  - Data on exposure obtained from parents (telephone interview)  - Indication for antibiotic therapy not reported | Typically developing Lebanese individuals without ASD (based on parental report) of the same age group, randomly selected from Greater Beirut area. | Diagnosis of cases performed by an experienced American Board of Neurology and Psychiatry licensed pediatric neurologist using the DSM-IV or DSM-V. Data obtained from the American University of Beirut Medical Center Special Kids Clinic. | The odds of having ASD after prenatal antibiotic exposure compared to unexposed - results from logistic regression analysis:  - bivariate analysis - OR 2.7 (95% CI 1.3-5.5)  - multivariable analysis - aOR 0.61 (95% CI 0.064–5.8) | Patients diagnosed with PDD-NOS and Asperger syndrome were excluded from the study. |
| Grossi 2018  Italy | - Autism group 1 (children with autism) n = 73  Autism group 1A  (subgroup of children with autism with healthy siblings), n=35  - Control group 1 (healthy children) n=96  - Control group 2 (healthy siblings of children with autism)  n=45  - Participants’ mean age (years): 8.2 (cases), 8.9 (controls) | - Early postnatal antibiotic exposure (0-3 months of age).  - Data on exposure obtained from mothers (structured interview)  - Indication for antibiotic therapy not reported | Children and adolescents without symptoms related to autism or learning disabilities living in the same area - mainly the study center’s staff relatives matched for age and possibly gender. | Independent diagnoses of autism according to DSM-V criteria, then confirmed by a qualified child and adolescent psychiatrist. | The odds of having autism after early postnatal antibiotic exposure compared to unexposed:  - autism group 1 vs. control group 1 - OR 2.43 (95% CI 0.9-6.52)  - autism group 1A vs control group 2 (siblings) - OR 2.03 (95% CI 0.40 – 9.1)  The main model was multivariable modelling of data performed with use of artificial neural network (more details in the main text). | Relatively small sample size. |
| Isaksson 2017  Sweden | - Children with ASD n = 206  - Controls n = 209  - Participants age: 4 - 10 years | - Antibiotics use prenatally and during breastfeeding  - Data on exposure reported by parents through web survey  - Indication for antibiotic therapy not reported | Siblings without ASD and healthy, unrelated children, all identified based on parental report.  (10% of controls had neurodevelopmental disorders other than ASD) | Cases identified based on parental report on ASD diagnosis. No other information on definition used or method of ASD diagnosis reported. | Prenatal maternal use of antibiotics:  ASD group: 16.9% vs non-ASD group: 14.0%  Maternal use of antibiotics during breastfeeding:  ASD group: 13.8% vs non-ASD group: 13.5%  Autism regressed with antibiotic use during pregnancy and breastfeeding. Logistic regression betas presented in the form of log odds.  Antibiotics during pregnancy: β -0.15 (SE 0.45, p>0.05) in regular regression, β 1.78 (SE 1.04, p>0.05) in fixed effects regression.  Antibiotics during breastfeeding: β 0.17 (SE 0.38, p>0.05) in regular regression, β -0.65 (SE 0.60, p>0.05) in fixed effects regression. | The primary focus of the study was the association of GI problems with ASD. Diagnoses of autism, Asperger’s syndrome,  and PDD-NOS were combined with ASD. |
| Mrozek-Budzyń 2013  Poland | - Children with autism n = 96  - Controls n = 192  - Participants age: 2-15 years. | - Prenatal antibiotic exposure  - Data on exposure obtained from parents (structured interview)  - Indication for antibiotic therapy not reported | Children without autism diagnosis (the first 2 children, who visited the general practitioner after the autistic child visit and met entry criteria served as controls). | Children with childhood or atypical autism (ICD10 - F84.0 or F84.1) diagnosed by child psychiatrists, based on data obtained from medical records from a psychiatric outpatient clinic. | Percent of cases vs. percent controls exposed to prenatal antibiotics: 17% vs. 2%, respectively (p<0.001). Only univariate analysis performed for antibiotic exposure. |  |
| Niehus 2006  USA | - Children with autism/ASD n = 75  - Controls n = 24  - Participants mean age: 11.6 years (cases); 9.4 years (controls) | - Antibiotic use before the age of 2 years.  - Data on exposure obtained from medical records.  - Indication for antibiotic therapy not reported | Typically developing children of the same age, based on parental report and physician’s records | Diagnosis of autism, PDD-NOS, or Asperger’s syndrome from a site clinician according to the ADI-R and ADOS criteria. Cases identified based on medical records. | Children with autism/ASD were found to use significantly more antibiotics than the typically developing children.  Mean number of antibiotics taken before the age of 2 years:    - 7.04 (SD 5.35) for ASD group without clinical regression (p<0.05).  - 6.62 (SD 4.90) for ASD with clinical regression (statistically not significant).  - 6.88 (SD 5.15) for autism/ASD group (p<0.01).  - 3.48 (SD 2.68) for the control group. | Small sample size. |

**Abbreviations:** ADHD, attention-deﬁcit/hyperactivity disorder; ASD, autism spectrum disorders; ADI-R, Autism Diagnostic Interview Revised; ADOS, Autism Diagnostic Observation Schedule; aHR, adjusted hazard ratio; CARS, childhood autism rating scale; CI, confidence interval; DSM-IV, Diagnostic and Statistical Manual of Mental Disorders – IV; ICD, International Statistical Classification of Diseases and Related Health Problems; OR, Odds Ratio; SD, standard deviation; SE, standard error; PDD-NOS, pervasive developmental disorder not otherwise specified
