# Supplementary material for: Early Life Exposure to Antibiotics and Autism Spectrum Disorders: A Systematic Review
Source: J Autism Dev Disord. 2019 Jun 8;49(9):3866–76. doi: 10.1007/s10803-019-04093-y (PMC6667689; doi:10.1007/s10803-019-04093-y)
Supplement: Supplementary file 3 — Supplementary material 3 (DOCX 34 kb) [file 10803_2019_4093_MOESM3_ESM.docx]

| **Study ID** | **Study Population** | | **Exposure** | **Outcome** | **Results** | **Comments** |
| --- | --- | --- | --- | --- | --- | --- |
| Atladottir 2012  Denmark | - Population-based birth cohort of  96736 children, with 976 children having ASD and n= 342 with infantile autism  - Participants age: 8-14 years | | - Prenatal antibiotic exposure.  - Data on exposure obtained from mothers (structured telephone interviews)  - Indication for antibiotic therapy not reported | Diagnoses of ASD (ICD10 -F84.0 F84.1, F84.5, F84.8, and F84.9.), obtained from the Danish Psychiatric Central Register. All diagnoses assigned by psychiatrists. | Slightly increased risk of ASD/infantile autism after the use of various antibiotics anytime during pregnancy as compared to unexposed.  The risk of ASD (crude HR & aHR reported):  - Any antibiotic: HR 1.2 (95% CI 1.0-1.4), aHR 1.2 (95% CI 1.0-1.4)  - Penicillins: HR 1.2 (95% CI 1.0-1.5), aHR 1.3 (95% CI 1.0-1.6)  - Macrolides: HR 1.3 (95% CI 0.8-2.1), aHR 1.3 (95% CI 0.7-2.2)  - Sulfonamides: HR 1.5 (95% CI 1.1-2.2), aHR 1.5 (95% CI 1.0-2.2)  - Cephalosporins: HR 1.3 (95% CI 0.7-2.3), aHR 1.1 (95% CI 0.6-2.3)  The risk of infantile autism (only aHR reported):  - Any antibiotic: aHR 1.2 (95% CI 0.9-1.7)  - Penicillins: aHR 1.4 (95% CI 1.0-2.0)  - Macrolides: aHR 2.2 (95% CI 1.1-4.4)  - Sulfonamides: aHR 1.6 (95% CI 0.9-3.0)  - Cephalosporins: aHR 1.9 (95% CI 0.8-4.7)  Trimester-specific aHRs also available for penicillins and sulfonamides. | Multiple testing is an important limitation of the study – 106 comparisons were made (the few statistically significant associations observed could be chance findings). |
| Axelsson 2019  Denmark | - Nation-wide, register-based cohort of 677 403 children, including 8267 children with autism  - Participants age: up to 18 years | | - Antibiotic treatment during the first two years of life  - Data on exposure obtained from The Register of Medicinal Products Statistics  - Indication for antibiotic therapy not reported | Diagnosis of ASD extracted from the Danish The Psychiatric  Central Research  Register (ICD-10 codes F84.0, F84.1 F84.5 F84.8). | Risk of autism in children who had been exposed to antibiotics as compared to unexposed:  Between-within sibling model:  Penicillin: aHR 1.05 (95% CI 0.93, 1.18)  Broader-spectrum antibiotics: aHR 1.05 (95% CI 0.95, 1.16)  Stratiﬁed sibling model:  Penicillin: aHR 1.09 (95% CI 0.91, 1.29)  Broader-spectrum antibiotics: aHR 1.16 (95% CI 1.01, 1.36)  Standard model:  Penicillin: aHR 1.11 (95% CI 1.04, 1.19)  Broader-spectrum antibiotics: aHR 1.10 (95% CI 1.04, 1.16) |  |
| Hamad 2018  Canada | - Population-based birth cohort of 214,834 children, with 2965 children having ASD  - Participants age: 18 months - 18 years | - Postnatal antibiotic exposure during the first year of life  - Data on exposure obtained from Drug Program Information Network, which captures all prescription drug dispensation outside the hospital setting.  - Indication for antibiotic therapy not reported | | ASD diagnosis identified using claims from the hospital discharge abstracts (ICD-9 codes 299.0, 299.1, 299.8 or 299.9, or ICD-10 F84.0, F84.1, F84.3, F84.5, F84.8 or F84.9), physician visit (ICD-9 code 299) or an ‘ASD’ identifier in educational special needs funding database. | The adjusted analysis showed a trend towards a reduced risk of ASD in infants exposed to various antibiotics in their first year of lives, with significant associations observed in males and in those residing in urban areas when stratified by sex and region.  The risk of ASD in exposed compared to unexposed:  :  - Any antibiotic: HR 0.93 (95% CI 0.87-1.0), aHR 0.91 (95% CI 0.84-0.99)  - Any antibiotic (males): HR 0.86 (95% CI 0.79-0.93), aHR 0.91 (95% CI 0.83-1.00)  - Any antibiotic (urban region): HR 0.89 (95% CI 0.82-0.97), aHR 0.85 (95% CI 0.77-0.94)  - Penicillin: HR 0.93 (95% CI 0.86-1.01), aHR 0.92 (95% CI 0.84-1.00)  - Macrolides: HR 0.92 (95% CI 0.82-1.03), aHR 0.87 (95% CI 0.77-0.99)  - Other beta-lactams: HR 1.00 (95% CI 0.88-1.12), aHR 0.93 (95% CI 0.82-1.05)  - Other antibiotics: HR 0.89 (95% CI 0.76-1.05), aHR 0.92 (95% CI 0.77-1.09)  Secondary analysis based on a sibling-controlled design:  Any antibiotic: adjusted HR 1.03 (95% CI 0.86–1.23) | Number of antibiotic courses or cumulative duration on antibiotics was not associated with ASD risk.  Authors do not believe the observed association was clinically meaningful. |
| Wimberley 2018  Denmark | - Nation-wide, register-based cohort of 780,547 children, including 9352 children with autism  - Participants age: up to 16 years | - Childhood antibiotic exposure preceding the first diagnosis of autism  - Data on exposure obtained from Danish National Prescription Registry  - Indication for antibiotic therapy not reported | | Diagnosis of autism, extracted from the Danish Psychiatric  Central Research Register (ICD-10 F84.0, F84.1 F84.5 F84.8 F84.9) | Risk of autism in children who had been exposed to broad-spectrum antibiotics compared to unexposed children : aHR 1.29 (95% CI 1.17–1.43)  Risk of autism in children exposed to different classes of antibiotics compared to individuals who had never used any antibiotics:  Broad-spectrum antibiotics exposure: aHR 1.51 (95% CI 1.33–1.72)  Moderate-spectrum antibiotics exposure aHR 1.22 (95% CI 1.13–1.32)  Narrow-spectrum antibiotics exposure aHR 1.08 (95% CI 0.99–1.18) |  |

**Abbreviations:** ADHD, attention-deﬁcit/hyperactivity disorder; ASD, autism spectrum disorders; ADI-R, Autism Diagnostic Interview Revised; ADOS, Autism Diagnostic Observation Schedule; aHR, adjusted hazard ratio; CARS, childhood autism rating scale; CI, confidence interval; DSM-IV, Diagnostic and Statistical Manual of Mental Disorders – IV; HR, crude hazard ratio; ICD, International Statistical Classification of Diseases and Related Health Problems; OR, Odds Ratio; SD, standard deviation; SE, standard error; PDD-NOS, pervasive developmental disorder not otherwise specified.
