# Supplementary material for: Early Life Exposure to Antibiotics and Autism Spectrum Disorders: A Systematic Review
Source: J Autism Dev Disord. 2019 Jun 8;49(9):3866–76. doi: 10.1007/s10803-019-04093-y (PMC6667689; doi:10.1007/s10803-019-04093-y)
Supplement: Supplementary file 4 — Supplementary material 4 (DOCX 35 kb) [file 10803_2019_4093_MOESM4_ESM.docx]

Online Resource 5. Excluded Studies

| **Study ID** | **Reason for exclusion** |
| --- | --- |
| (Adams et al. 2007) | Statistical model not designed to study association between antibiotic use and ASD. |
| (Fallon 2005) | No control group. |
| (Fang et al. 2015) | Study on the association between infections and subsequent risk of ASD. |
| (Gao et al. 2015) | Data on antibiotics collected, but no numerical data reported. |
| (Grossi et al. 2016) | A pilot study to validate a method for a further study included in this review. |
| (Heilbrun et al. 2015) | Antibiotic use did not precede the diagnosis of autism. |
| (Hisle-Gorman et al. 2018) | Data on antibiotic use reported collectively with antifungals and antivirals. |
| (House et al. 2016) | Not clear whether antibiotic exposure preceded ASD diagnosis. |
| (Kohler-Forsberg et al. 2018) | Data on antibiotic exposure reported collectively with exposure to other anti-infective agents. |
| (Lydholm et al. 2018) | Data on autism reported collectively with other developmental disorders |
| (Maimburg and Vaeth 2006) | Data on antibiotics collected, but no numerical data reported. |
| (Maramara et al. 2014) | Data on antibiotics collected, but no numerical data reported. |
| (Schmitz et al. 2017) | Children with ASD not compared to healthy children. |
| (Vargason et al. 2018) | Study to explore association between GI symptoms and antibiotic use in children with ASD. |
| (Zerbo et al. 2015) | Data on antibiotics collected, but no numerical data reported. |

Abbreviations: ASD, autism spectrum disorders; GI, gastrointestinal.

References for excluded studies:

Adams, J. B., Romdalvik, J., Ramanujam, V. M., & Legator, M. S. (2007). Mercury, lead, and zinc in baby teeth of children with autism versus controls. *J Toxicol Environ Health A, 70*(12), 1046-1051, doi:10.1080/15287390601172080.

Fallon, J. (2005). Could one of the most widely prescribed antibiotics amoxicillin/clavulanate "augmentin" be a risk factor for autism? *Med Hypotheses, 64*(2), 312-315, doi:10.1016/j.mehy.2004.06.023.

Fang, S. Y., Wang, S., Huang, N., Yeh, H. H., & Chen, C. Y. (2015). Prenatal Infection and Autism Spectrum Disorders in Childhood: A Population-Based Case-Control Study in Taiwan. *Paediatr Perinat Epidemiol, 29*(4), 307-316, doi:10.1111/ppe.12194.

Gao, L., Xi, Q. Q., Wu, J., Han, Y., Dai, W., Su, Y. Y., et al. (2015). Association between Prenatal Environmental Factors and Child Autism: A Case Control Study in Tianjin, China. *Biomed Environ Sci, 28*(9), 642-650, doi:10.3967/bes2015.090.

Grossi, E., Veggo, F., Narzisi, A., Compare, A., & Muratori, F. (2016). Pregnancy risk factors in autism: a pilot study with artificial neural networks. *Pediatr Res, 79*(2), 339-347, doi:10.1038/pr.2015.222.

Heilbrun, L. P., Palmer, R. F., Jaen, C. R., Svoboda, M. D., Perkins, J., & Miller, C. S. (2015). Maternal Chemical and Drug Intolerances: Potential Risk Factors for Autism and Attention Deficit Hyperactivity Disorder (ADHD). *J Am Board Fam Med, 28*(4), 461-470, doi:10.3122/jabfm.2015.04.140192.

Hisle-Gorman, E., Susi, A., Stokes, T., Gorman, G., Erdie-Lalena, C., & Nylund, C. M. (2018). Prenatal, perinatal, and neonatal risk factors of autism spectrum disorder. *Pediatr Res*, doi:10.1038/pr.2018.23.

House, S. A., Goodman, D. C., Weinstein, S. J., Chang, C. H., Wasserman, J. R., & Morden, N. E. (2016). Prescription Use among Children with Autism Spectrum Disorders in Northern New England: Intensity and Small Area Variation. *J Pediatr, 169*, 277-283.e272, doi:10.1016/j.jpeds.2015.10.027.

Kohler-Forsberg, O., Petersen, L., Gasse, C., Mortensen, P. B., Dalsgaard, S., Yolken, R. H., et al. (2018). A Nationwide Study in Denmark of the Association Between Treated Infections and the Subsequent Risk of Treated Mental Disorders in Children and Adolescents. *JAMA Psychiatry*, doi:10.1001/jamapsychiatry.2018.3428.

Lydholm, C. N., Kohler-Forsberg, O., Nordentoft, M., Yolken, R. H., Mortensen, P. B., Petersen, L., et al. (2018). Parental Infections Before, During, and After Pregnancy as Risk Factors for Mental Disorders in Childhood and Adolescence: A Nationwide Danish Study. *Biol Psychiatry*, doi:10.1016/j.biopsych.2018.09.013.

Maimburg, R. D., & Vaeth, M. (2006). Perinatal risk factors and infantile autism. *Acta Psychiatr Scand, 114*(4), 257-264, doi:10.1111/j.1600-0447.2006.00805.x.

Maramara, L. A., He, W., & Ming, X. (2014). Pre- and perinatal risk factors for autism spectrum disorder in a New Jersey cohort. *J Child Neurol, 29*(12), 1645-1651, doi:10.1177/0883073813512899.

Schmitz, J. C., Cholemkery, H., Medda, J., & Freitag, C. M. (2017). [Pre- and perinatal risk factors in autism spectrum disorder and attention deficit/hyperactivity disorder]. *Z Kinder Jugendpsychiatr Psychother, 45*(3), 209-217, doi:10.1024/1422-4917/a000507.

Vargason, T., McGuinness, D. L., & Hahn, J. (2018). Gastrointestinal Symptoms and Oral Antibiotic Use in Children with Autism Spectrum Disorder: Retrospective Analysis of a Privately Insured U.S. Population. *J Autism Dev Disord*, doi:10.1007/s10803-018-3743-2.

Zerbo, O., Qian, Y., Yoshida, C., Grether, J. K., Van de Water, J., & Croen, L. A. (2015). Maternal Infection During Pregnancy and Autism Spectrum Disorders. *J Autism Dev Disord, 45*(12), 4015-4025, doi:10.1007/s10803-013-2016-3.
