# Supplementary material for: Early Life Exposure to Antibiotics and Autism Spectrum Disorders: A Systematic Review
Source: J Autism Dev Disord. 2019 Jun 8;49(9):3866–76. doi: 10.1007/s10803-019-04093-y (PMC6667689; doi:10.1007/s10803-019-04093-y)
Supplement: Supplementary file 5 — Supplementary material 5 (DOCX 25 kb) [file 10803_2019_4093_MOESM5_ESM.docx]

Online Resource 6. Risk of Bias Summary for Cohort Studies

| **Quality Assessment Criteria** | **Criterion to be Fulfilled to Award Asterix (*)** | **Atladottir et al.** | **Hamad et al.** | **Axelsson et al.** | **Wimberley et al.** |
| --- | --- | --- | --- | --- | --- |
|  |  | **Selection (4*maximum)** | | | |
| **Representativeness of the exposed cohort** | a) truly representative of the average children in the community *  b) somewhat representative of the average children in the community *****  c) selected group of users eg nurses, volunteers  d) no description of the derivation of the cohort | * | * | * | * |
| **Selection of the non exposed cohort** | a) drawn from the same community as the exposed cohort *****  b) drawn from a different source  c) no description of the derivation of the non exposed cohort | * | * | * | * |
| **Ascertainment of exposure** | a) secure record (eg surgical records)^1^ *****  b) structured interview *****  c) written self report  d) no description | * | * | * | * |
| **Demonstration that outcome of interest was not present at start of study** | a) yes *****  b) no | * | * | * | - |
|  |  | **Comparability (2*maximum)** | | | |
| **Comparability of cohorts on the basis of the design or analysis** | 1. The study controls for clearly described confounding factors** 2. No control for, or no adequate description of confounding factors, | ** | ** | ** | ** |
|  |  | **Outcome (3* maximum)** | | | |
| **Assessment of outcome** | a) independent blind assessment *****  b) record linkage^2^ *****  c) self report  d) no description | * | * | * | * |
| **Was follow-up long enough for outcomes to occur?** | a) yes *****  b) no | * | * | * | * |
| **Adequacy of follow-up of cohorts** | a) complete follow up - all subjects accounted for *****  b) subjects lost to follow up unlikely to introduce bias - small number lost - <20% follow up, or description provided of those lost *****  c) follow up rate < 20% and no description of those lost  d) no statement | * | * | * | * |

Adapted from Newcastle-Ottawa scale (Retrieved from <http://www.ohri.ca/programs/clinical_epidemiology/oxford.asp>)

^1^Studies with information on exposure based on prescriptions/hospital records were considered as low risk of bias
^2^Data on ASD diagnosis from at least two independent sources, or from a source that was previously validated was treated as low risk of bias

Online Resource 7. Risk of Bias Summary for Case-Control Studies

| **Quality Assessment Criteria** | **Criterion to be Fulfilled to Award Asterix (*)** | **Issakson et al.** | **Mrozek-Budzyn et al.** | **Niehus et al.** | **Guisso et al.** | **Grossi et al.** | **George et al.** | **Bittker et al.** |  |
| --- | --- | --- | --- | --- | --- | --- | --- | --- | --- |
|  |  | **Selection (4*maximum)** | | | | | | | |
| **Is the case definition adequate?** | a) yes, with independent validation *****  b) record linkage or based on self reports  c) no description | **-** | **-** | ***** | **-** | ***** | **-** | **-** |  |
| **Representativeness of the cases** | a) consecutive or obviously representative series of cases *****  b) potential for selection biases or not stated | ***** | ***** | **-** | **?** | ***** | ? | ***** |  |
| **Selection of Controls** | a) community controls *****  b) hospital controls  c) no description | ***** | * | ? | ***** | **?** | ***** | ***** |  |
| **Definition of Controls** | a) no history of disease (endpoint) *****  b) no description of source | ***** | ***** | ***** | ***** | ***** | **-** | ***** |  |
|  |  | **Comparability (2*maximum)** | | | | | | | |
| **Comparability of cases and controls on the basis of the design or analysis** | a) The study controls for clearly described confounding factors**  b) No control for, or no adequate description of the confounding factors | ******. | **-** | **-** | ****** | ** | **-** | ****** |  |
|  |  | **Exposure (3* maximum)** | | | | | | | |
| **Ascertainment of exposure** | a) secure record (eg surgical records) *****  b) structured interview where blind to case/control status *****  c) interview not blinded to case/control status  d) written self report or medical record only  e) no description | **-** | **-** | ***** | **-** | **-** | **-** | **-** |  |
| **Same method of ascertainment for cases and controls** | a) yes *****  b) no | ***** | ***** | ***** | ***** | ***** | ***** | ***** |  |
| **Non-Response rate** | a) same rate for both groups^3^ *****  b) non respondents described  c) rate different and no designation | **?** | **?** | n/a^4^ | **-** | **?** | ***** | **-** |  |

Adapted from Newcastle-Ottawa scale (Retrieved from <http://www.ohri.ca/programs/clinical_epidemiology/oxford.asp>)

^1^Studies with information on exposure based on prescriptions/hospital records were considered as low risk of bias
^2^Data on ASD diagnosis from at least two independent sources, or from a source that was previously validated was treated as low risk of bias
^3^“Same rates” were defined as not significantly different between the groups (Fisher’s exact test, significance threshold = 0,05)
^4^Data was derived from medical records
