# Supplementary material for: Early Life Exposure to Antibiotics and Autism Spectrum Disorders: A Systematic Review
Source: J Autism Dev Disord. 2019 Jun 8;49(9):3866–76. doi: 10.1007/s10803-019-04093-y (PMC6667689; doi:10.1007/s10803-019-04093-y)
Supplement: Supplementary file 6 — Supplementary material 6 (DOCX 17 kb) [file 10803_2019_4093_MOESM6_ESM.docx]

| **Study ID** | **Confounding factors used for adjustment** |
| --- | --- |
| Atladottir 2012 | Gender, maternal age, parity, and maternal smoking during pregnancy, paternal age parental psychiatric history and parents’ educational status |
| Axelsson 2019 | Maternal age at birth, parental age difference, parental education, maternal marital status, maternal smoking, infant sex, 5-min Apgar score, use of CPAP or a ventilator, asphyxia, parental epilepsy, preeclampsia or hypertension, gestational diabetes, parity, maternal antibiotic use during the pregnancy, maternal infections during the pregnancy, parental psychiatric history |
| Bittker 2018 | Gender, age of the child, ethnicity, region (Midwest, South), maternal education, age of the mother at the time of birth of the child, relationship to the child |
| George 2014 | Confounding factors not described (refers reader to another study, which cannot be found) |
| Guisso 2016 | Gender, age, birth order, blood type, gestational period, type of delivery, birth weight, complications during delivery, jaundice, breastfeeding, feeding difficulty, infections during pregnancy, fever, medication, travel, pregnancy complications, folic acid intake, psychological support, alcohol, smoking, parents’ age, maternal blood type, parents’ work and education, history of psychiatric disease, consanguinity |
| Grossi 2018 | Semantic connectivity map model – no typical adjusted analysis performed |
| Hamad 2018 | Sex, region, health care access, SES, maternal age at delivery, maternal medical conditions (mood and anxiety disorders, schizophrenia, diabetes), prenatal infections, prenatal antidepressants use, prenatal smoking/drug/alcohol use, size for gestational age, childhood medical conditions (epilepsy, infections, neonatal jaundice, asthma and diagnosis with other developmental disability disorder), birth complications, mode of delivery, multiple birth, breastfeeding initiation, year of birth, season of birth and birth order |
| Isaksson 2017 | Age, sex, birth weight, and weight gain in pregnancy |
| Mrozek-Budzyń 2013 | Not adjusted |
| Niehus 2006 | Not adjusted |
| Wimberley 2018 | Sex, age, maternal, and paternal age at birth, gestational age, parental psychiatric disorders prior to birth, somatic hospitalization in the previous year and for parity (i.e., stratiﬁed by parity and calendar year). |

CPAP, continuous positive airway pressure; SES, socioeconomic status
